# Improving ANFO: Effect of Additives and Ammonium Nitrate Morphology on Detonation Parameters

**DOI:** 10.3390/ma14195745

**Published:** 2021-10-01

**Authors:** Magdalena Fabin, Tomasz Jarosz

**Affiliations:** 1 Faculty of Chemistry, Silesian University of Technology, 44-100 Gliwice, Poland; magdfab819@student.polsl.pl; 2 Department of Physical Chemistry and Technology of Polymers, Silesian University of Technology, 44-100 Gliwice, Poland

**Keywords:** ANFO, additives, detonation velocity, ammonium nitrate, fuel oil

## Abstract

Ammonium nitrate–fuel oil (ANFO) is one of the most widely used explosives for civilian purposes. Its main advantages are its low price and simple method of production. The main disadvantages of this material are low water resistance and problems related to non-ideal detonation, which can be a potential hazard when using ANFO. Due to this, research has been conducted for many years to find suitable additives for ANFO that would have the effect of offsetting its drawbacks. The aim of this review was to describe factors affecting the energetic properties of ANFO, including the highlighting of substances that could potentially be additives to ANFO formulations that would reduce the negative effects of non-ideal detonation, while avoiding adversely impacting the effectiveness of the explosive in blasting operations, as well as its sensitivity parameters.

## 1. Introduction

Ammonium-nitrate-based explosives are a broad class of explosive mixtures, the main component of which is ammonium nitrate, which also serves as the oxidising agent [1]. The most frequently used ammonium-nitrate-based materials include the following: ANNMAL, ammonal [2], amatol [3], and ANFO (Table 1). ANFO has been used since the mid-20th century [4]. The main advantages of ANFO include, among others, a low production cost and an uncomplicated production process.

ANFO belongs to the group of non-ideal high explosives (NIHEs), which differ from conventional explosives, as these explosives are characterised by high porosity and low density, as well as by the fact that the fuel and the oxidising agent are not combined in a single molecule and can form separate phases. As a result, NIHE-class materials are usually characterised by low detonation-velocity values [5]. The above-mentioned feature is the main problem of ANFO-type explosives, as low detonation velocities may distort and disrupt the process of controlled detonation. This in turn can lead to potentially hazardous issues with ANFO usage and storage, as well as reducing the efficiency of the explosive. That is why, over the course of many years, attempts have been made to supplement ANFO with additives that might reduce the problems related to the consequences of non-ideal detonation [6]. These attempts have focused mainly on replacing diesel oil with other types of oils; e.g., from recycled materials. Despite the fact that research demonstrated, among other things, an improvement in ANFO blasting properties, the use of recycled fuel oil (FO) substitutes leads to a possible risk of environmental contamination [7,8,9,10].

In this review, we summarised the most relevant recent developments in ANFO supplementation aimed at improving ANFO detonation properties and at eliminating the problems related to nonideal detonation.

## 2. Properties of ANFO

ANFO usually consists of 94 wt% porous AN, which acts both as an oxidising agent and adsorbent for the FO, which constitutes 6 wt% of ANFO [11].

**Figure 1:**
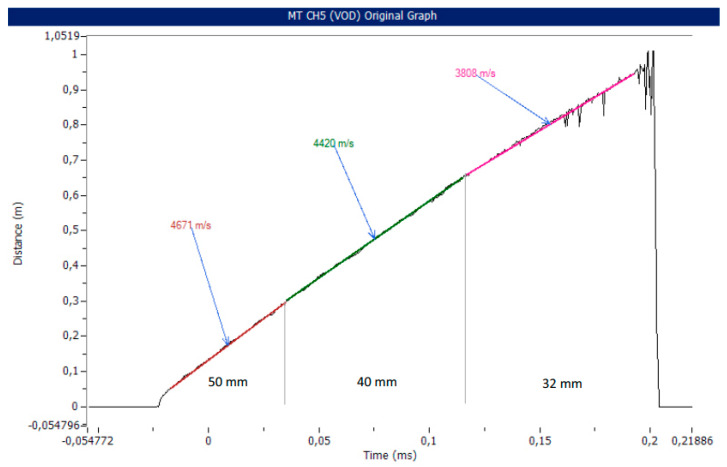
The influence of charge diameter on the velocity of detonation plot for an Emulinit 8 L sample tested in a sewage pipe of varying diameter. Reprinted with permission of the Łukasiewicz Institute of Industrial Organic Chemistry from [13].

NIHEs such as ANFO are the opposite of ideal high explosives (HEs), for which the detonation velocity exceeds the speed of sound velocity. When the diameters of NIHE charges are comparable to the limiting diameter for that NIHE, initiation by explosive shock is observed to disrupt the porous structure of the NIHE charge. Depending on the degree of this disruption, the achievable detonation velocity can be limited, or the NIHE charge may be incapable of sustaining detonation [12] (Figure 1).

For smaller charge diameters, the detonation velocity may differ from the ideal value that is expected based on equilibrium chemical thermodynamics. This difference is particularly pronounced for insensitive high explosives, and in individual cases can reach up to 30% of the ideal detonation velocity [14]. This is due to the fact that when detonating charges are of a finite diameter, the detonation front cannot be flat. In fact, due to the limited nature of the chemical transformation rate, immediately behind a flat detonation wave front, the flow is subsonic [15]. Therefore, the waves that are created by the lateral scattering of matter penetrate the original front, reducing the pressure on it and thus reducing the speed of the reaction wave front. In this way, the shock front of the detonation wave acquires a convex shape in the direction of the detonation propagation [15].

ANFO is a type of explosive that, despite its low detonation pressure and detonation velocity, is characterised by a significant destructive power due to the large volume of detonation gases [16]. ANFO is one of the most widely used explosives in the mining industry, despite competing in the market with the much more efficient emulsion explosives (EEs). This is mainly due to its uncomplicated manufacturing technology, small number of its components, and low production costs compared to EEs [17] (Table 2).

## 3. Modification of ANFO Properties

### 3.1. Modification of AN

Pure ammonium nitrate (AN) is considered to be a highly stable and relatively safe compound. AN is used mainly in agriculture as a nitrogen-rich fertilizer [21]. Global AN production was estimated at a level of 16.6 million metric tons in 2019 [22].

**Figure 2:**
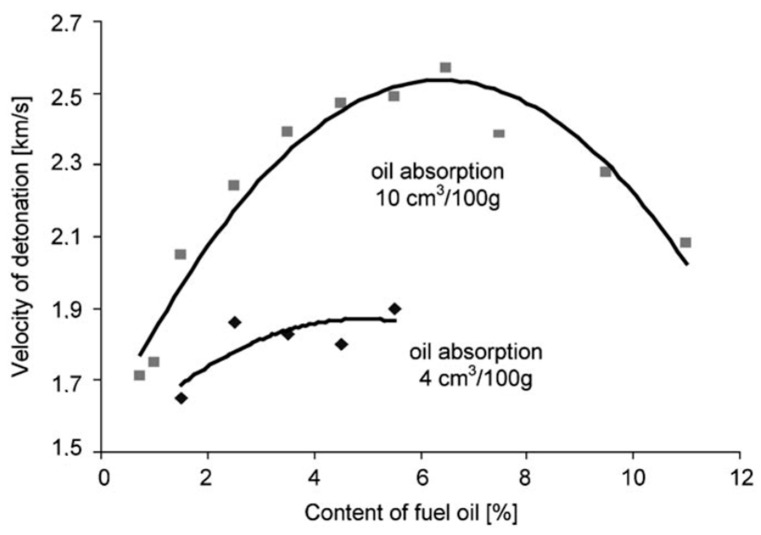
Detonation velocity of ANFO vs. content of fuel oil. Reprinted with the permission of John Wiley and Sons from [25].

AN is used as a component of many explosives, such as ANNM, amatols, and ammonals. However, in combination with fuel oils, it is used to create relatively safe ANFO explosives, with properties similar to the remaining explosive types [23,24]. When AN is used for the manufacturing of explosives, its porosity is one of the most important parameters. This property is associated with the effectiveness of the material as an adsorbent, which is particularly important in the case of AN/mineral oil mixtures (Figure 2).

**Figure 3:**
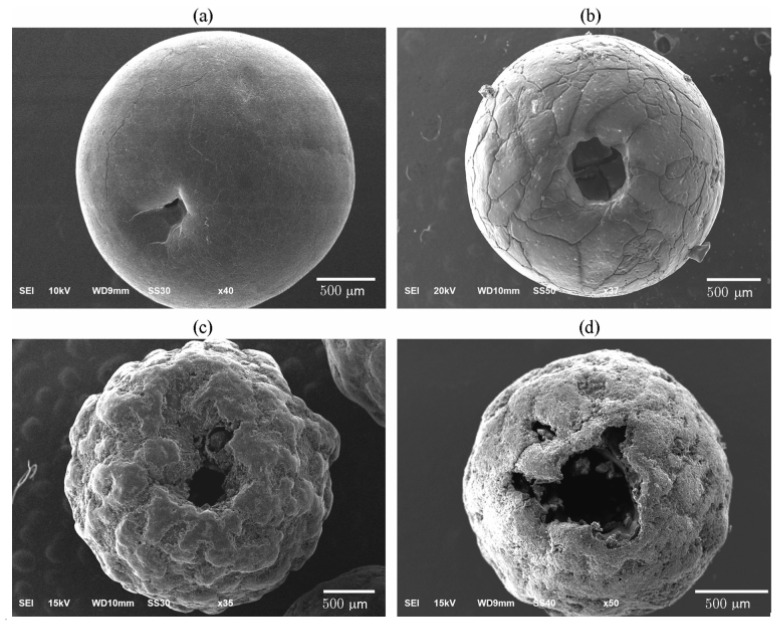
View of the outer surface of ammonium nitrate granules on the side of the shrinkage cavity: (**a**) GOST No. 2-2013 AN; (**b**) the same AN after thermal treatment (PorAN); (**c**) porous AN from China (PAN-MH); (**d**) porous AN from France (PAN-GP). Reprinted with the permission of Springer Nature from [26], copyright 2016.

Higher porosity increases the adsorption capacity of oils on the AN surface while reducing the density of the explosive [26,27] (Figure 3).

However, if the density of the material falls below a predetermined critical density value, detonation will not occur because it will not provide an adequate path for the propagation wave in the charge [13].

Fuel oil (FO) adsorption to an oil content of 15 cm^3^/100 g AN was accompanied by a decrease in AN density of approximately 35% [25]. Simultaneously, ANFO detonation velocity increased by over 70%. ANFO charges composed of AN with fuel oil absorptivity of less than 2.5 cm^3^/100 g failed to detonate under the testing conditions [25,28]. The minimum value of fuel oil absorptivity of AN granules at which full charge detonation was observed achieved was ca. 2.5–3.0 cm^3^/100 g, and the measured detonation velocity amounted to a value of 1.6–1.7 km/s (Table 3). With the value of fuel oil absorptivity achieving a level of 12–15 cm^3^/100 g, the detonation velocity achieved a maximum value of 2.7 km/s. During the testing procedure, there was no observed trend of a further increase in detonation velocity associated with the increase of the FO amount [25].

Studies conducted later also showed that there was a relationship between the amount of ground and granular AN in the ANFO composition and the detonation velocity of the explosive.

Under measured conditions, it was shown that the greatest increase in detonation velocity occurred when the content of granular AN was changed from 10 wt% to 15 wt%. A further increase in the content of granular AN had little effect on the detonation velocity [29] (Table 4 and Table 5).

The research also demonstrated that the manner in which porous AN was prepared was important for the ANFO parameters. As a result of constant manufacturing-process development, the granulated material undergoes a polymorphic change and proper product drying ensures its low moistness. This allows long-term storage of the product without losing its durability and properties [30,31].

In an attempt to increase ANFO sensitivity, the compatibility of a series of inorganic salts and organic substances with AN was investigated via differential scanning calorimetry. It was established that the salts of weak acids and urea increased the thermal stability of the mixtures in comparison with pristine AN, even in the presence of destabilizing components [32].

Reaction activation energy is one the most important AN parameters that can be influenced by additives. This is related to the reaction of AN decomposition. Research confirmed that alkali metal chlorides and alkaline earth metals demonstrated similar activation energy, while the lowest combustion pressure was achieved with an AN/NaCl mixture. In addition, the constant combustion rates of AN/NaCl and AN/BaCl_2_ mixtures were higher that the decomposition rate of pure AN [33]. The influence of calcium chloride on AN was also examined in earlier research. It was established that CaCl_2_ is a good anticaking agent [34].

Particular attention was paid to the influence of Na_2_SO_4_ salt and KCl salt on the AN decomposition. The results achieved for AN supplemented with 2.8 wt% of Na_2_SO_4_ demonstrated that the reaction initiation temperature increased by ca. 50 °C, and the temperature measured at the maximum spontaneous heating rate increased by ca. 34 °C in comparison with pure AN. This demonstrated that Na_2_SO_4_ additive may increase the AN decomposition reaction initiation temperature, thus acting as an inhibitor [35].

In the case of an AN/KCl mixture, the research demonstrated that the reaction initiation temperature was lower compared to the pure AN decomposition initiation temperature. Thus, it could be concluded that potassium chloride was a catalytic agent of the AN decomposition reaction [35].

Then, the influence of both salts on AN was tested. The research demonstrated that the combination of the AN decomposition inhibitor and its catalyst promoter increased the reaction initiation temperature, inhibiting its course and at the same time increasing pressure and the maximum spontaneous heating temperature, thus failing to stop the decomposition reaction. It was suspected that the inhibiting SO_4_^2−^ ion influence and catalysing influence of chloride anions overlapped [36] (Table 6). In addition, the research confirmed that the alkali metal chlorides and ammonium chloride reduced the temperature of the AN decomposition reaction initiation in a similar range. However, the results did not confirm any improvement of the explosive properties of AN-based explosives [37] (Table 7).

The problem of the amount of evolved gaseous products in the reactions of the thermal decomposition of AN mixed with other additives was also of interest. In the case of mixtures of 2AN/CaCO_3_, 2AN/MgCO_3_, and 2AN/0.5CaMg(CO_3_)_2_, the analysis indicated that at a constant temperature and while keeping the amount of AN constant at 2 mol, the equilibrium amount of evolved gases such as NO, NO_2_, or N_2_O did not depend on the content of carbonates and the type of carbonate added [38]. The equilibrium concentration of the mentioned gases varied with the running temperature of the process. In the case of the addition of CuSO_4_ to the mixtures of AN with carbonates, the concentration of nitrogen oxides did not change. However, the higher the temperature of the analyses, the higher the equilibrium concentration of sulphur oxides [33].

In the case of adding CuSO4(s), the CuO content increased to a value of 10^−7^–10^−5^ mol. In the case of adding H_3_BO_3_, MnO_2_, or CuSO_4_, the concentration of solid products such as CuO, Cu_2_O, or MnO, and gas products such as SO_2_, SO_3_, and HBO, reached the same level [37].

The combination of AN and explosives was also investigated. When combusting mixtures of AN with 15% methylnitrotetrazol (MNT) and 13.3% poly(glycidyl azide) (GAP), the low-pressure combustion limits of the FO-free mixtures occurred at pressures of 6–10 MPa. However, the combustion rates of these mixtures were quite low, and the reaction activation energy for the TNT addition was the highest of all the substances tested [33].

The research on inorganic salt additives concentrated not only on the added salt type, but also on the process of preparing the mixtures. It was demonstrated that in the case of mixtures obtained by mechanical mixing, the detonation velocity of such mixtures was reduced with the increase in the additive proportion. In the case of an AN/10% KCl mixture, its detonation velocity was reduced from a value of 1.78 km/s to a value of 1.43 km/s, and in the case of AN/15% NH_4_H_2_PO_4_ mixture, the detonation velocity was reduced to a value of 1.26 km/s. This confirmed the assumption that physical properties of additives had a significant influence on the properties of explosives. However, in the case of AN mixtures and the above-mentioned additives in a solution, the detonation velocity of the mixture increased to the following values: in the case of AN/5% KCl, 1.86 km/s; and in the case of AN/10% NH_4_H_2_PO_4_, 2.00 km/s [39].

The bulk density of mixtures prepared by solution mixing was lower than that of mixtures prepared by mechanical mixing. This means that the particles became smaller while the porosity became larger. This proved that mixtures with AN obtained by different mixing methods had different particle sizes, bulk densities, and surface morphologies, which affected their explosive properties [39] (Table 8).

Due to a recent increase in the interest in polymers, it was decided to coat the porous AN with long-chained LS50% soya resin. The results of field-emission scanning electron microscopy (FE-SEM) and nuclear magnetic resonance (NMR) spectroscopy did not demonstrate drastic differences between the modified and pure AN. Modified AN passed a water-resistance test. Unfortunately, after preparing an ANFO sample out of the substance, no detonation was observed [40].

### 3.2. Fuel Modification and Alternative Fuels

Fuel oil is an important component of ammonium-nitrate-based explosives. In the case of ANFO, the constituents include, inter alia, diesel oil, kerosene, or gasoline. Research conducted on diesel oil enriched with FTC/FPC combustion catalytic agents demonstrated that the catalytic agents used did not influence the ANFO’s explosive properties [41]. Additionally, the fuel oil pyrolysis also did not significantly influence the detonation velocity. As a result of the detonation of ANFO achieved by mixing AN with fuel oils obtained by pyrolysis, the obtained medium explosion energy value was in the range of (P1–P5) 3950–4020 J/g, which was close to the energy of an ANFO reference sample explosion; i.e., 3940 J/g [42].

Due to a lack of fuel additives that could significantly influence ANFO explosivity, it was attempted to replace fuels composed of long-chain alkanes with other organic substances. It was observed that a mixture of AN with a biodiesel fuel produced satisfactory results compared to a typical ANFO mixture. A mixture of ANFO and rice straw produced the worst result, with an average detonation velocity of ca. 1759.2 m/s. For the remaining tested ground fuels (corn cob, extracted sugarcane, and tyre remnants) the average detonation velocity fell within the range of 1759.2 m/s to 3.177 m/s [43].

Other research concentrated on examining the detonation velocity along with the measurement of the reaction initiation temperature for mixtures of AN with vegetable substances. The studies demonstrated that all of the tested mixtures displayed distinct explosive properties. Their detonation velocities were lower than ANFO, but still within a range of 2–3 km/s [44] (Table 9).

Spent lubricating oils (LOs) are a relatively problematic waste due to their environmental harmfulness. One proposed method of dealing with such waste products was to use them for the preparation of ANFO in place of typical fuel oils [45]. Testing revealed that although the use of such oils instead of typical fuel oils had a slight adverse effect on the energetic properties of ANFO; when no more than 20 wt% of LO was added to the diesel oil used to produce ANFO, no significant influence on detonation parameters of the material was observed [46] (Table 10).

### 3.3. Additives to ANFO

Due to a problem of low sensitivity, nonideal detonation, and low water resistance, research was conducted over the course of many years aimed at finding additives to ANFO that could eliminate the above-mentioned problems. The costs of blasting procedures with the use of ANFO usually are from one-half to one-third of the costs of the same blasting procedures conducted with emulsion-type, slurry-type, and even cartridge-type explosives. ANFO is also relatively easy to produce [47].

The problem lies in inducing and ensuring the continuity of the detonation of ANFO charges. In order to improve detonation parameters, tests were conducted on additives added directly to explosives. Aluminium dust is one of the most frequently tested additives [29,48,49,50]. The test demonstrated that depending on the amount of the added dust in wt%, the total concentration of toxic gas products of the ANFO detonation reaction can be reduced by half. The exothermic aluminium combustion reaction mainly supports the process of dissociation and disintegration of NO, NO_2_, and NO_x_ radicals. An increase in the amount of Al particles from 3.6% to 12% also led a 1.7-fold decrease in hydrocarbon content (oil) from 4.51 to 2.65 wt% [46]. The form of added aluminium also had an influence on detonation velocity.

The conducted research demonstrated that after exceeding the 10 wt% amount of the aluminium additive, the ANFO detonation velocity decreased (Table 5).

Other additives that have attracted the attention of scientists are bio-waste products. It was determined that the optimum content proportion, defined as AN:fuel oil:bio-waste, amounted to 90:5:5. This proportion demonstrated higher absorption and physical stability than the other mixtures tested. The modified ANFO demonstrated an increase in detonation velocity, from a value of 2300 to a value of 3800 m/c, and an increase in the brisance coefficient from a value of 20.5 to a value of 27.5, as compared to ordinary ANFO [51].

Research was also conducted on coating the AN used for ANFO preparation with silicon oxide (IV). It was demonstrated that while small amounts of SiO_2_ could be used to reduce the amount of postblasting fumes and slightly increased the energetic properties of ANFO, increasing the amount of added SiO_2_ resulted in rapid deterioration of the performance of ANFO charges [52].

## 4. Discussion and Conclusions

Based on the analytical results of the conducted tests, it was concluded that the addition of alkali and alkaline earth metal chlorides increased the detonation velocity, while alkali metal sulphates slowed down the propagation wave of the detonation reaction. Moreover, chlorides decreased the reaction start temperature.

The method of preparation of additives affected the bulk density and grain size of the explosive, which translated into a change in detonation parameters; e.g., VOD.

The morphology and grain size distribution of the AN used to prepare ANFO are important factors that determine the energetic parameters of the resultant explosive formulation. Most existing works have focused on studying the effect of those parameters on the achieved detonation velocity, rather than the full set of energetic properties. Nevertheless, even when only considering the aspect of detonation velocity, it could be noted that the frequently mentioned empirical rule of “higher density results in higher detonation velocity” was not necessarily true for ANFO, and a more comprehensive approach should be adopted. It is worth noting that, depending on the adsorption features of the AN, a higher ANFO density may translate into a lower achieved detonation velocity.

The addition of aluminium to ANFO accelerated the detonation reaction, and depending on the form of the additive used, different detonation velocities could be obtained. Adding a mixture of FO and other fuels to ANFO, such as biodiesel or lubricants, decreased the detonation velocity, reaction pressure, and amount of released gases. The substitution of FO with plant-derived substances such as wheat flour, milled rapeseed, or wood dust resulted in a lower VOD, but it was still within the explosive range of ANFO.

Coating AN to increase wood resistance did not yield the expected results for ANFO’s explosive parameters due to oxidant desensitisation. However, this opened the way for the agricultural industry towards a safeguard against the use of agricultural AN for high-energy materials.

Research conducted over the years showed that some of the additives did not return satisfactory results for improving the detonation performance of ANFO. This was mainly related to the problems of a nonideal detonation mechanism. The introduction of organic compounds with high carbon content to the explosive in relation to its nitrogen or oxygen content led to a decrease in detonation velocity, or even inhibited the detonation process.

Ammonium-nitrate-based additives attract constant attention. On the basis of the demonstrated research, we cannot explicitly say that the additives tested up to this point can eliminate the problems related to nonideal detonation. The impact of specific substances on ANFO detonation parameters is very limited, and while they may impact one of the parameters in a positive manner, they may have a negative impact on the other parameters. Mixing various additives may generate extra production costs and may fail to impact detonation parameters in a satisfactory manner. Newly tested additives are expected to improve ANFO’s properties by changing its detonation parameters in a manner that will eliminate the disadvantages of ANFO-type materials.

## Figures and Tables

**Table 1:** Comparison of detonation composition and detonation velocity in commonly used, industrially prepared ammonium-nitrate-based materials [2,3,4].

Parameter	Explosive		
	ANFO	ANNMAL	Amatol
Composition (wt%)	AN-94 FO-6	AN-66 NM(nitromethane)-25 Al-5 C-3 TETA (triethylenetetramine)-1	AN-50 TNT-50
Density (kg/m^3^)	917.86	1158.13	1496.12
Typical detonation velocity (km/s)	5269.93	5359.94	6289.91

**Table 2:** Comparison of energetic properties of typical ANFO and EE formulations [17,18,19,20].

Parameter	ANFO	EE
Critical diameter (mm)	50–80	16–46
Loading density (g∙cm^−3^):	0.75–0.85	0.90–1.20
Detonation model:	Non-ideal	Ideal (provided it contains no stable components)
Detonation velocity (m∙s^−1^):	1800–3300	3800–5100
Water-resistance	No	Yes
Components	Ammonium nitrate (>90 wt%), FO (1–10) wt%	Oxidising agents, organic fuels, inorganic fuels, water, emulsifying agents, sensitizing agents, modifying agents
Manufacturing technology	Uncomplicated	Complicated
Price	Low	High
Trauzl lead block test (cm^3^/10 gPb)	211.83	360
Ballistic mortar test (%)	51.09	80.4–84.4

**Table 3:** Detonation velocity of ANFO prepared from AN granules of different sizes. Reprinted with the permission of John Wiley and Sons from [25].

Granule Size (mm)	ANFO Density (g/cm^3^)	Detonation Velocity (m/s)
1.00–1.20	1.02	No detonation
0.63–1.00	1.01	2330
0.50–0.63	1.02	2500
0.20–0.50	0.98	2960
0.20–0.50	0.86	3440

**Table 4:** Comparison of detonation velocities for different ANFO compositions [29].

ANFO Symbol	Weight ANFO Composition (%)			Density (g/cm^3^)	Detonation Velocity (m/s)
	AN_granular porous_	AN_milled_	Fuel Oil		
ANFO-10	10	84.5	5.5	0.88	1210
ANFO-15	15	79.5		0.90	2200
ANFO-20	20	74.5		0.90	2360
ANFO-30	30	64.5		0.92	2570
ANFO-40	40	54.5		0.93	2660
ANFO-50	50	44.5		0.97	2680
ANFO-60	60	34.5		0.98	2620

**Table 5:** Detonation velocity of ANFO with the addition of aluminium [29].

Lp.	ANFO Composition (wt%)				Density (g/cm^3^)	Detonation Velocity (m/s)	
	AN_granular porous_	AN_milled_	FO	Al		Al_atomized_	Al_flaked_
1	44.5	50	5.5	-	0.97	2680	2680
2	42.3	47.5	5.2	5	0.97	2680	2710
3	40.1	45.0	4.9	10	0.96	2990	2840
4	35.6	40.0	4.4	20	0.88	2400	2300
5	31.2	35.0	3.8	30	0.83	ND	1490

**Table 6:** Effect of Na_2_SO_4_ and KCl on AN parameters. Reprinted with the permission of Elsevier from [35,36].

Experimental Results for Pure AN						
NH_4_NO_3_(g)		T_onset_ (°C)	P_onset_ (MPa)	(dT/dt)_max_ (°C s^−1^)	(dP/dt)_max_ (MPa s^−1^)	T_max_ (°C)
3.5		190	1.59	65	1.1	318
		200	1.45	82	1.43	343
		210	1.45	98	2.02	381
Average		200 (±10)	1.50 (±0.1)	82 (±17)	1.52 (±0.50)	347 (±42)
**Experimental Results for AN/Na_2_SO_4_ Mixture at Different Concentrations**						
**Na_2_SO_4_ wt%**	**Na_2_SO_4_ (g)**	**T_onset_ (°C)**	**P_onset_ (MPa)**	**(dT/dt)_max_ (°C s^−1^)**	**(dP/dt)_max_ (MPa s^−1^)**	**T_max_ (°C)**
1.13	0.04	240 (±7)	1.45 (±0.02)	166 (±12)	3.50 (±0.44)	394 (±4)
1.69	0.06	248 (±7)	1.46 (±0.03)	119 (±20)	3.39 (±0.36)	387 (±6)
2.78	0.1	250 (±10)	1.45 (±0.02)	115 (±16)	2.74 (±1.13)	381 (±4)
5.41	0.2	255 (±5)	1.45 (±0.03)	132 (±22)	2.92 (±1.05)	392 (±8)
10.26	0.4	263 (±4)	1.48 (±0.05)	113 (±19)	1.65 (±0.21)	379 (±12)
12.50	0.5	268 (±1)	1.47 (±0.03)	107 (±8)	1.57 (±0.10)	388 (±5)
36.36	2	276 (±2)	1.54 (±0.12)	19 (±17)	0.36 (±0.14)	377 (±10)
**Experimental Results for KCl Mixture at Different Concentrations**						
**KCl wt%**	**KCl(g)**	**T_onset_ (°C)**	**P_onset_ (MPa)**	**(dT/dt)_max_ (°C s^−1^)**	**(dP/dt)_max_ (MPa s^−1^)**	**T_max_ (°C)**
2.78	0.1	194 (±2)	1.41 (±0.08)	332 (±35)	5.39 (±1.92)	301 (±2)
5.41	0.2	196 (±3)	1.43 (±0.03)	290 (±65)	4.77 (±2.11)	323 (±18)
7.89	0.3	196 (±2)	1.43 (±0.01)	373 (±32)	10.02 (±2.43)	309 (±10)
11.39	0.45	180 (±5)	1.42 (±0.01)	420 (±20)	8.87 (±0.84)	292 (±6)
12.50	0.5	152 (±9)	1.40 (±0.01)	490 (±96)	7.82 (±2.03)	295 (±15)
22.22	1	145 (±8)	1.39 (±0.01)	503 (±65)	7.36 (±1.83)	302 (±3)
**Experimental Results for AN/Na_2_SO_4_/KCl Mixture at Different Concentrations**						
**Na_2_SO_4_ + KCl**		**T_onset_ (°C)**	**P_onset_ (MPa)**	**(dT/dt)_max_ (°C s^−1^)**	**(dP/dt)_max_ (MPa s^−1^)**	**T_max_ (°C)**
**Mass (g)**	**Mol%**					
0.25 + 0.25	3.61 + 6.87	231 (±3)	1.50 (±0.01)	468 (±86)	1.50 (±0.01)	344 (±35)
0.5 + 0.5	6.53 + 12.44	237 (±3)	1.50 (±0.01)	637 (±144)	1.50 (±0.01)	343 (±1)
0.5 + 0.0	7.45 + 0	268 (±1)	1.60 (±0.10)	107 (±8)	1.60 (±0.10)	388 (±5)
0.0 + 0.5	0 + 13.31	152 (±9)	1.50 (±0.10)	596 (±92)	1.50 (±0.10)	295 (±15)

**Table 7:** Effect of chloride on AN parameters studied by MCPVT. Reprinted with the permission of Elsevier from [37].

Sample	Chloride Concentration in Samples	T_onset_ (°C)	P_onset_ (kPa)
AN	-	280	44.82
AN-BaCl_2_	0.1%	228	30.34
	0.5%	222	34.47
	1%	215	32.41
AN-NH_4_Cl	0.5%	231	37.23
	2%	216	77.91
AN-CaCl_2_	0.1%	226	31.03
	0.5%	220	32.41
	1%	213	37.23
AN-NaCl	0.1%	231	33.78
	0.5%	213	35.85
AN-KCl	0.1%	230	40.68
	0.5%	224	45.51
	1%	215	53.09

**Table 8:** Differences in bulk densities and grain sizes depending on the method of mixture preparation. Reprinted with the permission of Springer Nature from [39].

Sample	Mixing Method	Bulk Density (g/cm^3^)	Grain Size (μm)
AN/KCl	In solution	0.68	420–841
AN/KCl	Mechanically	0.76	250–420
AN/NH_4_H_2_PO_4_	In solution	0.55	177–250
AN/NH_4_H_2_PO_4_	Mechanically	0.70	149–177

**Table 9:** Measurements of explosivity parameters for pure AN and mixtures of AN with vegetable substances. [44].

Material	Composition of Materials (wt%)		Decomposition Temperature (°C)		Critical Diameter (mm)	Detonation Velocity (km/s)
			Start	Onset		
AN	-		ca.203	ca.250	45	1.65
ANFO	AN 94.5	Fuel oil 5.5	203	227	35	3.56
ANWF	AN 85	Wheat flour 15	165	170	45	3.14
ANHC	AN 90	Coal 10	182	196	40	2.84

**Table 10:** Properties of ANFO explosives based on lubricating oil. Reprinted with permission of John Wiley and Sons from [46].

Properties	ANFO	20 wt% LO in D/O	30 wt% LO in D/O	40 wt% LO in D/O	50 wt% LO in D/O
Density (g/cc)	0.79	0.78	0.79	0.79	0.8
VOD (m/s)	4579	4537	4375	4247	4137
Detonation pressure (GPa)	4.14	4.01	3.78	3.56	3.42
SO_2_ (g) (um/m^3^)	7.95	7.12	6.98	8.11	7.19
NO_x_ (g) (um/m^3^)	18.2	18.2	15.6	16.5	14.9
Particulate matter 2.5 (um/m^3^)	42.6	42.4	44.9	42.9	45.9
Particulate matter 10 (um/m^3^)	64.2	63.5	66.3	65.9	63.7
